# Impact of patient knowledge on hypertension treatment adherence and efficacy: A single-centre study in Poland

**DOI:** 10.7150/ijms.48139

**Published:** 2021-01-01

**Authors:** Anna Paczkowska, Karolina Hoffmann, Krzysztof Kus, Dorota Kopciuch, Tomasz Zaprutko, Piotr Ratajczak, Michał Michalak, Elżbieta Nowakowska, Wiesław Bryl

**Affiliations:** 1 Department of Pharmacoeconomics and Social Pharmacy, Poznan University of Medical Sciences. Rokietnicka 7, 60-806 Poznan, Poland.; 2 Department of Internal Diseases, Metabolic Disorders and Arterial Hypertension, Poznan University of Medical Sciences. Szamarzewskiego 84, 60-569 Poznan, Poland.; 3 Department of Computer Science and Statistics, Poznan University of Medical Sciences Rokietnicka 7, 60-806 Poznan, Poland.

**Keywords:** hypertension, prevention, patient education, adherence to medical recommendations, treatment efficacy

## Abstract

**Introduction:** Recent studies show that treatment of arterial hypertension is unsuccessful. This is due to the patients' insufficient knowledge of about the therapeutic methods and the consequences of not treating arterial hypertension.

**Objectives:** The aim of the study was to evaluate the patients' knowledge concerning therapeutic options, prophylaxis, and complications of arterial hypertension. The study also assessed the effect of such knowledge on hypertension treatment adherence and efficacy.

**Patients and Methods:** The survey included 488 patients (250 female and 238 male), aged over 18 years, diagnosed with and treated in outpatient and inpatient settings at selected healthcare institutions in Poland. A custom-made questionnaire, based on references on this subject, was the key tool in the present study. Information about the course of the disease and evaluation of hypertension treatment efficacy was based on the patients' medical records.

**Results:** The study found that 54.7% of the subjects had good knowledge about arterial hypertension, 40.0% had average knowledge, and 5.3% had poor knowledge. The extent of knowledge about the disease was significantly dependent on the level of education and the place of receiving medical care (*p*< 0.05). Good knowledge was significantly associated with controlled blood pressure, number of antihypertensive drugs used, frequency of hospitalization, as well as with medication adherence, and healthy lifestyle behaviours (*p*< 0.05).

**Conclusions:** More than half of the patients presented good knowledge but a large group still had poor knowledge, especially patients with a low level of education and with hypertension treated at a general practitioner's clinic. The results of our study clearly show that knowledge about arterial hypertension affects medication adherence and healthy lifestyle behaviours and improves hypertension treatment efficacy.

## Introduction

Arterial hypertension represents a serious medical, social, and economic problem in Poland. According to the latest Multi-center National Population Health Examination Surveys - WOBASZ study 1,2, epidemiological situation concerning arterial hypertension in Poland has deteriorated. It is estimated that in the years 2013-2014, the age-standardized prevalence, awareness, treatment, and control of hypertension was 42.7%, 59.3%, 46.1%, and 23%, respectively 2.

Recent studies show that hypertension treatment is unsuccessful mostly due to the lack of cooperation between patients and physicians in terms of lifestyle changes and compliance with the prescribed pharmaceutical therapy 2. This situation results from the fact that patients do not receive sufficient information about therapeutic methods and the consequences of failure to treat or improper treatment of their disease 3. Moreover, a number of studies show that the vast majority of patients (70-90%) have insufficient knowledge concerning therapeutic options in arterial hypertension and the related risks. Consequently, the patients do not trust their physicians and often deny the necessity of treatment if there are no symptoms 4. The lack of patient cooperation decreases their chances of reducing the total risk of cardiovascular diseases by even 40% 5.

Scientific literature does not offer sufficient data on the effect of patient knowledge on hypertension treatment adherence and efficacy in Poland.

The primary aim of the study performed was to assess the effect of patient knowledge on adherence to medical recommendations and improving efficacy of hypertension treatment. Another aim was to identify sociodemographic factors (age, gender, education level) and clinical factors (disease duration and place of providing medical care) that significantly affect the level of patient knowledge concerning hypertension.

## Materials and Methods

### Study population

The prospective, cross-sectional survey was conducted at the leading provincial hypertension clinic in Poland (Poznan city) over the period of one year (from 1 January 2019 to 31 December 2019). In the study, 565 patients (all patients treated for hypertension in the studied time horizon - from 1 January 2019 to 31 December 2019, who agreed to participate in the study) were taken into consideration. However, based on the inclusion and exclusion criteria of the study and incomplete filled in questionnaires by 77 patients, 488 patients were eventually included in the study. The target group of 488 patients (250 female and 238 male), aged over 18 years old, diagnosed with and treated in outpatient and inpatient settings for primary hypertension were selected by their attending physicians to participate in the study based on the inclusion and exclusion criteria described below.

It is estimated that about 11 million patients in Poland suffer from hypertension 6. Taking into account the size of the patient population and the significance level of α = 0.05 and the permissible estimation error e = 5%, the minimum number of the sample size was n = 385, therefore the study target group of 488 patients is representative for the hypertensive population. Prior to surveying, each of the eligible candidates was informed about the study objective and conditions, and gave their written informed consent to participate.

The main inclusion criteria were: adult age of the patients (>18 years old), arterial hypertension diagnosed according to the ICD-10 classification, diagnosed arterial hypertension treated in the studied time horizon (from 1 January 2019 to 31 December 2019), and patient's consent to participation in the study. On the other hand, the main exclusion criteria were: the lack of the patient's consent to participate in the study and diagnosed arterial hypertension treated for a different time period.

### Study technique

The key tool in the present study was a custom-made questionnaire based on references on this issue. The questionnaire consisted of 20 closed-ended questions related to the level of patient knowledge regarding therapeutic options, prevention, risk factors for hypertension and complications of hypertension, and 5 questions regarding treatment adherence (compliance with antihypertensive drug regime, frequent blood pressure measurements, dietary sodium chloride restriction, body weight control, regular physical activity). Moreover, specific information about the patients was collected by means of 13 demographic questions (about age, gender, level of education, place of living, source of income, place of receiving medical care, body mass index). The questions were based on professional references and then submitted for evaluation by national medical consultants in the field of hypertension. In order to validate and assess the accuracy and “comprehensibility” of the questionnaire, it was pre-tested on a sample of 150 patients (representative sample) with hypertension to whom the purpose of the study was explained. Subsequently, the questions could potentially be revised. Results of the pre-test were included in the final study since the pre-test did not yield any major modifications of the instrument. Information about the course of the disease and evaluation of hypertension treatment efficacy was based on the patients' medical records: duration of the disease, current average daily blood pressure values (24-Hour Ambulatory Blood Pressure Monitoring) measured by nurses during outpatient and inpatient treatment, hypotensive medications used, and frequency of hypertension-related hospitalisation.

In accordance with the latest guidelines of the American Heart Association/American College of Cardiology 2017 (AHA/ACC 2017) and European guidelines, the ESC/ESH 2018 values below 130/80 mm Hg were adopted as target blood pressure values 7. All the patients filled the questionnaires independently. Complete questionnaires (100% filled in by the patients) were included in the survey. The method of handling the information obtained during the survey guaranteed absolute confidentiality since every patient was identified as N.N; thus, the research project was in compliance with the Personal Data Protection Act.

The study protocol was approved by the Ethics Committee of the Poznan University of Medical Sciences.

### Statistical analysis

Quantitative parameters were presented using mean value, the median, and standard deviation. Categorical data were presented as counts and percentages. Results obtained for individual study groups were compared using the t-student test. If the data did not follow the normal distribution (the Shapiro-Wilk test), the comparison was performed using the Mann-Whitney test. Comparison of more than two groups was performed using the Kruskal-Wallis test with post hoc Dunn's test. The chi-square test for independence was used to analyse categorical data.

The relationship between the knowledge level and study parameters was analysed using the multiple regression analysis. For categorical data, the coefficient for a specified level was compared to the reference level.

The analysis was performed with the use of statistical package TIBCO Software Inc. (2017). Statistica (data analysis software system), version 13. http://statistica.io. All tests were considered significant at *p*<0.05.

## Results

### Study group characteristics

The study group included 488 people: 238 men (48.8%) and 250 women (51.2%), aged 63.7 ± 13 years. Higher education was declared by 55 subjects (11.2%), secondary by 214 subjects (43.9%), vocational by 175 subjects (35.9%), and primary by 44 subjects (9%) (Table 1).

Mean BMI value was 30.0 ± 5.9 kg/m^2^. A vast majority of the respondents were obese (62.7%) were treated for arterial hypertension on an outpatient basis at specialist hypertension clinics (79.1%), and 15.8% of the study participants required hospitalisation for blood pressure normalisation. Mean value of disease duration was 13.2 ± 9.3 years. Mean value of systolic blood pressure for the study's total population was 141.4 ± 13.3 mmHg, while diastolic blood pressure was 86.3 ± 11.4 mmHg. Most of the patients (97.6%) received polytherapy with 2.6 ± 1.0 drugs (mean ± SD) (Table 2). As a result of the applied treatment, 18.2% of the participants achieved the desired degree of blood pressure normalization (blood pressure values below 130/80 mmHg) (Table 2).

### Evaluation of knowledge regarding arterial hypertension

The level of knowledge regarding arterial hypertension was evaluated based on 20 questions in which the patients were asked about the diagnosis, therapeutic options, risk factors, as well as prevention and complications of the condition. The respondents were awarded 1 point for every correct answer. The total score represented patient knowledge regarding arterial hypertension. It was assumed that poor, average, and good knowledge about arterial hypertension was represented by a total score of 1 to 12, 13 to 16, and 17 to 20 points, respectively.

Mean level of knowledge of all the patients was 16.4 ± 2.4. It was found that 54.7% of the subjects had good knowledge about arterial hypertension, 40.0% had average knowledge, and 5.3% had poor knowledge (Table 3).

Statistical analysis carried out using the Kruskal-Wallis test and the chi-square test for independence showed that gender, age of the respondents, duration of the disease, and the respondents' body mass index had no effect on their level of knowledge on arterial hypertension (Table 3).

Statistical analysis carried out using the Kruskal-Wallis test showed that the patients with a higher education level had a better knowledge on arterial hypertension (65.5% of patients with good knowledge) than those with vocational education (48.6 of patients with good knowledge). The differences found were statistically significant (*p* = 0.0034) (Table 4).

Statistical analysis carried out using the Kruskal-Wallis test showed that the patients who were treated at specialist inpatient hypertension clinics and hospital wards were significantly more knowledgeable about their disease (66.2% of patients with good knowledge) than the ones treated only at specialist outpatient hypertension clinics and at general practitioner offices: 55.7% and 4.0% of patients with good knowledge, respectively (*p* = 0.01) (Table 4).

In addition, the performed Multiple Regression analysis for confounders that may influence the level of knowledge regarding arterial hypertension found that knowledge level depended on the patient's education, source of income, and place of receiving medical care (*p*<0.05) (Table 5). Patients with higher education level, working, and treated at specialist inpatient hypertension clinics and hospital wards had the highest level of knowledge regarding hypertension. Age, BMI, place of residence, as well as the duration of the disease did not affect the patient`s level of knowledge (*p*>0.05) (Table 5).

The survey indicated that 84.1% of the arterial hypertension patients were interested in participating in an educational programme on prophylaxis, therapeutic options, and preventing complications of arterial hypertension; 11.4% of the subjects were not willing to participate in such a programme, while 4.5% had no opinion on the matter.

### The impact of patient education on the effectiveness of hypertension treatment

Results of the analyses revealed that the level of patient knowledge on hypertension had a significant effect on its improved treatment (*p*< 0.0001). In patients with good knowledge on arterial hypertension, the values of systolic and diastolic blood pressure were significantly (*p*< 0.0001) lower than in the patients with average or poor knowledge (132/78 mmHg, 148/93 mmHg, and 177/113 mmHg, respectively) (Table 6). The proportion of patients with blood pressure normalisation was the highest in patients with good knowledge on hypertension (33.0% of patients with blood pressure below 130/80 mmHg) (*p*< 0.0001) (Table 6).

Moreover, average number of hypotensive medications used in this group was also significantly (p<0.0001) lower (1.8) that in the other groups (3.3 among patients with average knowledge, 4.8 among patients with poor knowledge). The proportion of patients hospitalised in the previous year due to arterial hypertension was the lowest in the group with good knowledge regarding arterial hypertension (1.5%) (Table 6).

### The impact of patient education on treatment adherence

Study results have indicated that the level of patient knowledge on arterial hypertension had a significant effect on compliance. Patients with good knowledge significantly (*p*< 0.0001) more frequently took hypotensive drugs as ordered (99.6%) compared to patients with average (53.3%) and poor knowledge (3.9%) (Table 7).

A similar phenomenon was observed for adherence to non-pharmacological treatment in arterial hypertension. Regular physical activity and weight reduction diet were declared by 96.6% of the patients with good knowledge, only 6.2% of the patients with average knowledge, and none of the patients with poor knowledge (*p*< 0.0001) (Table 7). Regular blood pressure monitoring at home was declared by 98.9% of the patients with good knowledge, merely 17.4% of the patients with average knowledge, and none of the patients with poor knowledge (*p*< 0.0001) (Table 7). Dietary salt restriction was declared by 99.6% of the patients with good knowledge, 53.3 of the patients with average knowledge, and 3.9% of the patients with poor knowledge. The differences observed were statistically significant (*p*< 0.0001) (Table 7).

## Discussion

Results of the present study are the first reports about the effect of patient education regarding arterial hypertension on hypotensive treatment adherence and efficacy in Poland. Since hypertension is very widespread, the subject matter of the described results is very relevant and significant for both clinicians and healthcare policy makers. According to the latest statistical data, cardiovascular diseases remain the main cause of mortality in Poland and in Europe. In 2010, deaths due to these diseases accounted for ca. 40% of all deaths in the Polish population. Arterial hypertension is the most common risk factor for cardiovascular diseases 9. These diseases, however, can be effectively prevented by prophylactic measures, the most vital of which is patient health education. Its implementation is the responsibility of not only healthcare professionals that are in contact with patients, but predominantly of patients themselves as their health is their own responsibility. Patient awareness is based on good knowledge of not only factors predisposing to arterial hypertension but also of therapeutic options and complications of an inefficiently treated disease 10.

The study target group (488 patients) was representative for hypertensive population in Poland. A vast majority of the respondents were female (51.2%), aged above 60 years. As a result of the treatment applied, 18.2% of the participants achieved the desired degree of blood pressure normalization (below 130/80 mmHg). The above study population characteristics are consistent with other properly conducted, epidemiological studies in Poland (WOBASZ, WOBASZ II) 1,2. A vast majority of the study group of WOBASZ II 1 were also women (51.7%), aged above 50 years. Hypertension control rates, in WOBASZ 2 and WOBASZ II 1 were also similar to those in the present study (23% and 24.3%, respectively).

Results of this study show that the arterial hypertension patients investigated have an extensive knowledge about their disease, but this is not sufficient. Over a half of the patients (54.7%) had good knowledge about arterial hypertension, while 40.0% had average knowledge, and 5.3% had poor knowledge. These results corroborate with the ones in the study carried out in Poland in 2014 by Wojciechowska M. et al 11. These authors also demonstrated that over a half of arterial hypertension patients in Poland presented with good knowledge, but there was still a large group of patients whose knowledge was poor. Metelska et. al. 12 reported that the knowledge on prevention of arterial hypertension of most surveyed patients was insufficient (43%) or sufficient (40%). Furthermore, studies conducted on 248 patients with hypertension from Central Poland revealed that the level of knowledge regarding hypertension was very poor 13. Most patients (79%) were unaware of the optimal blood pressure (BP) range. Elderly patients did not know the symptoms of hypertension (23.7%), were unwilling to make lifestyle changes (57%-65%), and had a poor awareness of hypertension therapy in the absence of symptoms (28.7%). The authors of the study conclude that efforts should be made to improve the knowledge of hypertension, especially among rural population, elderly patients, and those with a low-education level.

Our results also corroborate with those of a comprehensive study on a population of 2,500 patients, carried out by the Large Health Maintenance Organization in North Carolina 14. Results of that survey suggest that the knowledge of basic terminology used to communicate about hypertension and the awareness that hypertension is a risk factor for stroke and myocardial infarction is relatively good. Nevertheless, although the patients' knowledge about basic hypertension concepts appears to be good, the knowledge about blood pressure targets and current hypertension control status, particularly with respect to SBP, is suboptimal.

Extensive knowledge about arterial hypertension among patients was also shown in the US study by Viera A.J et al. 15 who, based on selected questions, evaluated the level of knowledge as relatively good. Approximately 44% of the respondents gave no incorrect answers, 34% answered incorrectly to 1 out of 12 items, and 22.3% answered incorrectly to 2 or more items. These results highlight the success achieved in high BP education over the last 30 years in the USA.

As the studies reported in the literature show, the level of patient knowledge about arterial hypertension varies across different nationalities. For example, approximately one-third of Turkish patients with arterial hypertension had poor knowledge about their disease, and very few patients (6.6%) had adequate knowledge 16. In Spain, approximately 92% of patients with arterial hypertension had inadequate knowledge about their disease, while very few patients (5.2%) had moderately good knowledge, and only 2.6% had good knowledge 17. All these data indicate that patients in developed counties have a better knowledge about arterial hypertension than people in underdeveloped or developing countries. It means that there may be a correlation between the degree of industrialisation and arterial hypertension awareness.

The multiple regression analysis of our study results shows that the general level of knowledge about arterial hypertension is significantly dependent on the level of education, source of income, and place of receiving medical care. Patients with a higher education level, working, and treated at specialist inpatient hypertension clinics and hospital wards had the highest level of knowledge regarding hypertension. Moreover, statistical analysis demonstrates that the level of knowledge about arterial hypertension is higher among women. The identified difference was, however, not statistically significant. The above-mentioned correlations are confirmed by other authors. A Polish study on the assessment of patient knowledge regarding prevention and complications of arterial hypertension, carried out by Sawicka et al. 18, also indicated that knowledge on the topic depended on gender and the level of education. Similarly to our study, women had a better knowledge about arterial hypertension than men, and persons with higher education had a better knowledge about arterial hypertension than persons with secondary or primary education.

Study results indicate that the level of patient knowledge on arterial hypertension is significantly dependent on the place of receiving healthcare. Patients receiving hypertension treatment in hospital settings or in specialist clinics were characterized by a significantly higher level of knowledge than patients treated by general practitioners. The study shows that hypertension treatment at specialist centers or clinics yields measurable therapeutic outcomes as patients receive comprehensive treatment; and patient education on prophylaxis, treatment and complications of inefficiently treated disease is a crucial element of the therapeutic process. These results are valuable for healthcare policy makers as they may be an incentive to increase the proportion of patients treated at specialized hypertension clinics where treatment efficacy is significantly higher. According to the latest report of the Polish National Health Fund (NFZ), in 2018 there were 20.4 million primary care consultations related with hypertension as the main or concomitant diagnosis. The respective numbers for specialist outpatient centers and inpatient treatment were: 2.8 million and 877,000. Compared to 2013, the number of hypertension-related primary care consultations decreased by approximately 6 million (over 23% compared to 2013), and the number of hospitalizations dropped by approximately 55,000 (nearly 6%). The number of consultations within specialist outpatient care, however, increased by approximately 605,000 (over 28%) 19.

The patients selected for the present study were highly interested in educational classes (84.1%). A significantly smaller proportion of study subjects were willing to exchange experience with other patients (40.3%). Our results corroborate with those reported by other investigators. In the study by Coulter et al. 20, a vast majority of patients were also very interested in receiving information about arterial hypertension. Patients put particular emphasis on their wish to be educated on the causes and consequences of the disease as well as the very essence of the disease. It must be clearly stressed that, as many authors agree, patients demonstrated a strong interest in finding out more about the disease despite suffering from chronic arterial hypertension and being under medical care of a highly specialised cardiac clinic. This shows that patients receive too little information during their routine appointments 21, 22.

Results of our study clearly show that knowledge about arterial hypertension affects medication adherence and healthy lifestyle behaviours. Patients with good knowledge significantly more frequently took hypotensive drugs as ordered (99.6%) compared to patients with average (53.3%) and poor knowledge (3.9%). A similar phenomenon was observed for adherence to non-pharmacological treatment methods in arterial hypertension (regular physical activity, weight reduction diet, salt restriction). Regular blood pressure monitoring at home was declared by 98.9% of the patients with good knowledge, merely 17.4% of the patients with average knowledge and none of the patients with poor knowledge. The results are in line with those reported by other authors. A study conducted in Poland by Jankowska-Polańska et al. 23 included 233 patients diagnosed with arterial hypertension and treated with hypotensive drugs for at least 1 year has shown that the patients' knowledge on hypertension is a significant independent determinant of good adherence. Other independent determinants include non-pharmaceutical treatment and regular blood pressure measurements. Akoko et al. 24 investigated 221 hypertensive patients in the Bamenda Health District, Cameroon, and showed that among 14% of the participants who had adequate knowledge of hypertension, 74.2% were compliant while 25.8% were non-compliant. Of the 86% of the participants who did not have adequate knowledge of hypertension, 38.9% were compliant while 61.1% were non-compliant. This relationship between compliance and knowledge on hypertension was statistically significant. Current results are in agreement with the study conducted by Malik et al. 25 where both drug adherence and BP control rates were significantly associated with knowledge about hypertension. A study that reviewed adherence to cardiovascular medications in developing countries, which was based on 76 studies, showed similar findings, namely that the most common predictors of poor drug adherence were poor knowledge, negative perceptions about the medication, side effects, and high medication cost 26. Many studies show that educational initiatives in hypertensive patients improve medication adherence and healthy lifestyle behaviours and decrease blood pressure levels 27, 28. In the study by Hacihasanoglu et al. 29, healthy lifestyle behaviours (weight control, salt restriction, regular physical activity) and perception of self-efficacy regarding medication adherence showed improvement after a six-month education programme carried out by a nurse.

Results of the present study demonstrated that the level of patient knowledge on arterial hypertension has a significant effect on treatment efficacy. The percentage of patients with controlled blood pressure was the highest (33.0%) among the patients with good knowledge regarding arterial hypertension. These results are innovative as they are the first reports showing that an appropriate level of patient education regarding their disease contributes to the normalization of blood pressure, thereby reducing the number of hypotensive drugs used and decreasing the number of hospitalizations related to uncontrolled hypertension per year. In the group of patients with good knowledge, the average number of hypertensive drugs was the lowest (1.8 ± 0.5) compared to other groups of patients (3.3 ± 0.5 for average knowledge, 4.8 ± 0.4 for poor knowledge). Moreover, the percentage of patients hospitalised during the past year was also the lowest (1.5%) compared to other groups (35.9% for average knowledge, 96.2 for poor knowledge). The above results are consistent with previous studies. A systematic review by the ISPOR Medication Adherence and Persistence Special Interest Group 30 established that improving patient knowledge regarding medications is of potential clinical value in improving adherence to antihypertensive therapy and interventions aimed at blood pressure control. Results of these studies support the notion that improving knowledge and gaining an understanding of the long-term risk of hypertension are valid approaches to improving hypertension treatment efficacy. Similarly, Canzanello et al. 31 reported significant decreases in blood pressure values in hypertensive individuals following an educational programme carried out by physicians and nurses in order to promote medication adherence and healthy lifestyle behaviours.

Overall, the present study along with the studies published earlier show that education positively affects medication adherence and healthy lifestyle behaviours and improves hypertension treatment efficacy.

Our study has some limitations, though. The most important limitation is the fact that this study sample was recruited from a single center. It would be very interesting to subsequently roll the study out onto other centers. Nevertheless, compared to previous studies in this field conducted in Poland 11, 12, 13, the presented results of the study covered a greater number of patients with arterial hypertension. Finally, no information on the patients' sources of knowledge was gathered. Considering the insufficient number of studies related to the effect of hypertensive patients' knowledge on adherence to medical recommendations and efficacy of hypertension treatment in Poland 23, however, this study might be recognized as an important contribution in the field.

## Conclusions

In conclusion, over a half of the patients presented with good knowledge but a large group still had poor knowledge. The latter applies in particular to the patients with low education and with hypertension treated at general practitioner clinics. A vast majority of arterial hypertension patients were willing to join educational programmes in spite of assessing their level of knowledge about the disease as sufficient.

Results of our study clearly show that knowledge about arterial hypertension affects medication adherence and healthy lifestyle behaviours and improves efficacy of hypertension treatment. Moreover, these research results constitute a valuable source of information for healthcare policy makers regarding the need to increase hypertension treatment at specialized clinics that effectively educate patients about their disease.

## Figures and Tables

**Table 1:** General characteristics of patients included in the evaluation of knowledge regarding arterial hypertension, n=488

Characteristics		
Group size	Total	488
	Female n (%)	250 (51.2)
	Male n (%)	238 (48.8)
Age	Total Mean ± (SD)	63.7 ± (13)
Education	Higher n (%)	55 (11.2)
	Secondary n (%)	214 (43.9)
	Vocational n (%)	175 (35.9)
	Primary n (%)	44 (9%)
Source of income	Work n (%)	118 (24.2%)
	Pension n (%)	357 (73.2%)
	Supported by parents n (%)	6 (1.2)
	Unemployed n (%)	7 (1.4)
Place of residence	Village n (%)	168 (34.4)
	Town of <10,000 inhabitants n (%)	63 (12.9)
	Town of 10,000-100,000 inhabitants n (%)	224 (45.9)
	City of 100,000-500,000 inhabitants n (%)	10 (2.1%)
	City of over 500,000 inhabitants n (%)	23 (4.7)

**Table 2:** Clinical characteristics of patients included in the evaluation of knowledge regarding arterial hypertension, n=488

Characteristic	
**Body mass index - BMI [kg/m^2^]**	
Mean ± (SD)	30.0 ± (5.9)
Normal body weight - BMI < 25 kg/m^2^, n (%)	98 (20.1)
Overweight - BMI 25-29.9 kg/m^2^, n (%)	84 (17.2)
Obesity - BMI ≥ 30 kg/m^2^, n (%)	306 (62.7)
**Duration of the disease (years), Mean ± (SD)**	13.2 ± (9.3)
**Blood pressure (mmHg)**	
Systolic, Mean ± (SD)	141.4 ± (13.3)
Diastolic, Mean ± ( SD)	86.3 ± (11.4)
**Controlled blood pressure (<130/80mmHg), n (%)**	89 (18.2%)
**Place of receiving medical care**	
Specialist clinic n (%)	386 (79.1)
Specialist clinic and hospital ward n (%)	77 (15.8)
General practitioner's office n (%)	25 (5.1)
**Number of hypertensive drugs, Mean ± (SD)**	2.56 ± (1.0)

**Table 3:** Sociodemografic and clinical factors influencing the level of knowledge regarding arterial hypertension, n=488

Variables	Level of knowledge regarding arterial hypertension			*P* value
	Poor knowledge	Average knowledge	Good knowledge	
General population (%)	5.3	40.0	54.7	
**Gender**				0.5086*
Women (%)	4.0	40.4	55.6	
Men (%)	6.7	39.5	53.8	
Age (Mean ± SD; Median)	61.5 ± 14.0; 65	63.5 ± 13.5; 66	64.2 ± 12.6; 63	0.5583**
Duration of the disease (Mean ± SD; Median)	10.5 ± 8.8; 7	13.4 ± 9.7; 10	13.5 ± 9.1; 12	0.2096 **
BMI (Body Mass Index) (Mean ± SD; Median)	30.4 ± 4.9; 29	30.0 ± 5.64; 29	29.9 ± 6.1; 29	0.7283**

*The chi-square for independence; **The Kruskal-Wallis test.

**Table 4:** Sociodemografic and clinical factors influencing the level of knowledge regarding arterial hypertension, n=488

Variables	Level of knowledge regarding arterial hypertension	*P* value*
Education: (Mean ± SD; Median)		0.0008
Primary	16.1 ± 2.8; 17^a,b^	
Vocational	16.0 ± 2.5; 16^a^	
Secondary	16.6 ± 2.2; 17^a,b^	
Higher	17.4 ± 1.9; 18^b^	
Place of receiving medical care: (Mean ± SD; Median)		<0.0001
Specialist clinic and hospital ward	17.2 ± 1.8; 18^a^	
Specialist clinic	16.4 ± 2.3; 17^b^	
General practitioner`s clinic	13.8 ± 3.0; 15^c^	

*The Kruskal-Wallis test; a,b,c - groups followed by the same letter do not differ statistically significantly.

**Table 5:** Multiple Regression analysis for the level of knowledge regarding arterial hypertension, n=488

Variable	Coeff.	Std. Err.	Standarized coeff.	t	*p*-value
**Gender**					
F					
M	-0.8	0.21	-0.02	-0.38	0.704
Age	0.02	0.01	0.09	1.35	0.176
Duration of disease	0.01	0.01	0.04	0.77	0.443
BMI	0.02	0.02	0.05	-0.40	0.319
**Education**					
Primary					
Vocational	-0.16	0.39	-0.03	1	0.691
Secondary	0.47	0.39	0.10	1.21	0.227
Higher	1.02	0.49	0.14	2.06	0.039
**Place of residence**					
Village					
Town of <10,000 inhabitants	-0.12	0.34	-0.02	-0.35	0.728
Town of 10,000-100,000 inhabitants	-0.04	0.24	-0.01	-0.16	0.877
City of 100,000-500,000 inhabitants	-0.67	0.77	-0.04	-0.88	0.379
City of over 500,000 inhabitants	0.43	0.53	0.04	0.81	0.418
**Source of income**					
Supported by parents					
Work	2.34	0.97	0.42	2.42	0.016
Unemployed	1.07	1.26	0.05	0.84	0.401
Pension	2.02	0.99	0.42	2.02	0.044
**Place of receiving medical care**					
Specialist clinic and hospital ward					
Specialist clinic	-0.75	0.33	-0.13	-2.26	0.025
General practitioner's clinic	-3.33	0.54	-0.31	-6.14	<0.001

**Table 6:** Assessment of the impact of the patient's level of knowledge on the effectiveness of hypertension treatment, n=488

Variables	Level of knowledge regarding arterial hypertension			*P* value
	Poor knowledge	Average knowledge	Good knowledge	
Systolic blood pressure (mmHg) (Mean ± SD; Median)	177.2 ± 15.4; 169^c^	148.8 ± 5.7; 148^b^	132.6 ± 6.0; 134^a^	< 0.0001*
Diastolic blood pressure (mmHg) (Mean ± SD; Median)	113.7 ± 6.4; 111^c^	93.8 ± 5.8; 94^b^	78.2 ± 5.4; 79^a^	< 0.0001*
Controlled blood pressure (<130/80 mmHg) (%)	0.0^a^	0.5^a^	33.0^b^	< 0.0001**
Number of hypertensive drugs (Mean ± SD; Median)	4.8 ± 0.4; 5^c^	3.3 ± 0.5; 3^b^	1.8 ± 0.5; 2^a^	< 0.0001*
Undergoing hospitalisation during the past year (%)	96.2^c^	35.9^b^	1.5^a^	< 0.0001**

*The Kruskal-Wallis test; **The chi-square for independence a,b,c - groups followed by the same letter do not differ statistically significantly.

**Table 7:** Assessment of the impact of the patient's level of knowledge on adherence to medical recommendations, n=488

Variables	Level of knowledge regarding arterial hypertension			*P* value*
	Poor knowledge	Average knowledge	Good knowledge	
Regular use of antihypertensive drugs (%)	3.9^a^	53.3^b^	99.6^c^	< 0.0001
Regular blood pressure monitoring (%)	0.0^a^	17.4^b^	98.9**	< 0.0001
Regular physical activity (%)	0.0^a^	6.2^a^	96.6^b^	< 0.0001
Weight reduction diet (%)	0.0^a^	6.2^a^	96.6^b^	< 0.0001
Dietary sodium chloride restriction (%)	3.9^a^	53.3^b^	99.6^c^	< 0.0001

*The chi-square for independence a,b,c - groups followed by the same letter do not differ statistically significantly.
